# 1644. Quantifying Empiric Vancomycin Overuse in Two Tertiary Care Pediatric Intensive Care Units

**DOI:** 10.1093/ofid/ofad500.1478

**Published:** 2023-11-27

**Authors:** Kathleen Chiotos, Jason Newland, Luke Starnes, Didien Meyahnwi, Rebecca G Same, Ebbing Lautenbach, Julia E Szymczak, Jeffrey S Gerber

**Affiliations:** Children's Hospital of Philadelphia, Philadelphia, PA; Washington University School of Medicine, Saint Louis, MO; Washington University in St. Louis, St. Louis, Missouri; Children's Hospital of Philadelphia, Philadelphia, PA; Children's Hospital of Philadelphia, Philadelphia, PA; University of Pennsylvania, Philadelphia, Pennsylvania; University of Utah, Salt Lake City, Utah; Children's Hospital of Philadelphia, Philadelphia, PA

## Abstract

**Background:**

Vancomycin is among the most commonly prescribed antibiotics in US children’s hospitals. When used empirically for sepsis, vancomycin primarily targets methicillin-resistant *Staphylococcus aureus* (MRSA). We aimed to quantify empiric vancomycin overuse among critically ill children with suspected sepsis.

**Methods:**

We performed a cross sectional study including all episodes of suspected sepsis in two tertiary care pediatric intensive care units (PICUs) between 11/2020 and 10/2022. Suspected sepsis was defined as collection of a blood culture and administration of one or more broad spectrum antibiotics within 12 hours. Episodes with prior broad-spectrum antibiotic use or transfers from an outside hospital within 7 days were excluded, as were episodes occurring within 14 days of a preceding sepsis episode. The frequency of MRSA among episodes of suspected sepsis was calculated based on the results of sterile site, respiratory, and wound cultures collected within 24 hours of the sepsis episode. MRSA history was defined as a positive clinical or screening culture or PCR within 6 months before the suspected sepsis episode. As a secondary analysis, we quantified the frequency of any vancomycin-requiring organism (Table 1).

**Figure:**
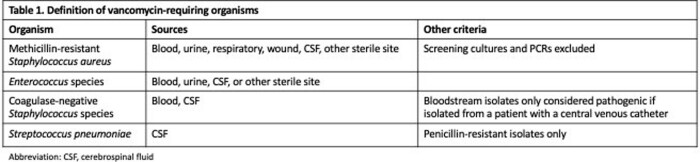

**Results:**

We identified 1859 suspected sepsis episodes. Of these, 1135 (61%) received empiric vancomycin for a median of 3 days (IQR 2,3). MRSA was identified in 37 (2%) and any vancomycin-requiring organism was identified in 101 (5%). Among 1790 episodes with no history of MRSA, 1080 (60%) received empiric vancomycin and MRSA was identified in 17 (< 1%). MRSA was infrequent in community onset sepsis (21/1324 episodes, 2%) and in patients without central lines (18/1397, 1%) (Table 2). The proportion of episodes receiving empiric vancomycin differed between the two centers (70% vs 44%, *P*< 0.01), as did the proportion with a history of MRSA (3% vs 5%, *P*=0.03), whereas identification of MRSA (1% vs 0.9%, *P*=.77) did not.

**Figure:**
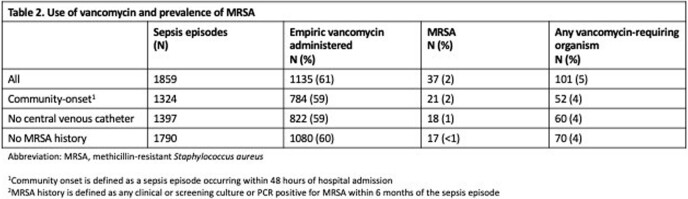

**Conclusion:**

Vancomycin was administered to 61% of critically ill children initiated on broad-spectrum antibiotics, but MRSA was identified in just 2%. Reducing empiric vancomycin overuse for community onset sepsis and in patients with no history of MRSA or central venous catheter is an actionable target for antimicrobial stewardship efforts in the PICU.

**Disclosures:**

**Jason Newland, MD**, Moderna: Grant/Research Support|Pfizer: Grant/Research Support

